# Brain EV‐Bound Proteins in Blood as a Robust Biomarker for Accurate Classification of Tau Tangles in the Brain

**DOI:** 10.1002/alz.088533

**Published:** 2025-01-09

**Authors:** Nicholas RY Ho

**Affiliations:** ^1^ Sunbird Bio, Singapore, Singapore Singapore

## Abstract

**Background:**

Brain derived Extracellular Vesicles (EVs) was isolated from blood and their containing pthogenic proteins were quantified to develop a biomarker signature for the classification of Tau aggregation in the brain.

**Method:**

Brain derived EVs and their cargo were measured directly in the plasma of patients with AD and determined to be Tau‐PET positive (visual read) (n=10) and other age matched healthy controls (n = 22) at one time‐point. AD pathogenic proteins bound to EVs were quantified by in‐house custom developed immunoassays.

**Result:**

A signature of a variety brain derived EV bound total tau, P‐T231‐tau, and P‐S396‐tau was able to accurately classify Tau‐PET positive AD patients from healthy controls (AUC: 0.88 – 0.93)

**Conclusion:**

Brain derived EV bound total tau, P‐T231‐tau, and P‐S396‐tau in blood show good potential as biomarkers to classify Tau aggregation in the brain

